# Influences of Additives on the Rheological Properties of Cement Composites: A Review of Material Impacts

**DOI:** 10.3390/ma18081753

**Published:** 2025-04-11

**Authors:** Ke Xu, Jie Yang, Haijie He, Jingjie Wei, Yanping Zhu

**Affiliations:** 1 Zhejiang Fangyuan New Materials Co., Ltd., Taizhou 318000, China; kexu0001@126.com; 2 School of Civil Engineering and Architecture, Taizhou University, Taizhou 318000, China; 3 Department Civil, Architectural and Environmental Engineering, Missouri University of Science and Technology, Rolla, MO 65401, USA; jwxhc@mst.edu; 4 Civil Engineering Department, Montana Technological University, Butte, MT 59701, USA; yzhu@mtech.edu

**Keywords:** rheological properties, supplementary cementitious materials, chemical admixtures, nanomaterials, cement composites

## Abstract

Cement-based materials are essential in modern construction, valued for their versatility and performance. Rheological properties, including yield stress, plastic viscosity, and thixotropy, play indispensable roles in optimizing the workability, stability, and overall performance of cement composites. This review explores the effects of supplementary cementitious materials (SCMs), chemical admixtures, nanomaterials, and internal curing agents on modulating rheological properties. Specifically, SCMs, including fly ash (FA), ground granulated blast furnace slag (GGBFS), and silica fume (SF), generally improve the rheology of concrete while reducing the cement content and CO_2_ emissions. Regarding chemical admixtures, like superplasticizers (SPs), viscosity-modifying agents (VMAs), setting-time control agents, and superabsorbent polymers (SAPs), they further optimize flow and cohesion, addressing issues such as segregation and early-age shrinkage. Nanomaterials, including nano-silica (NS) and graphene oxide (GO), can enhance viscosity and mechanical properties at the microstructural level. By integrating these materials above, it can tailor concrete for specific applications, thereby improving both performance and sustainability. This review presents a comprehensive synthesis of recent literature, utilizing both qualitative and quantitative methods to assess the impacts of various additives on the rheological properties of cement-based materials. It underscores the pivotal roles of rheological properties in optimizing the workability, stability, and overall performance of cement composites. The review further explores the influences of SCMs, chemical admixtures, nanomaterials, and internal curing agents on rheological modulation. Through the strategic integration of these materials, it is possible to enhance both the performance and sustainability of cement composites, ultimately reducing carbon emissions and advancing the development of eco-friendly construction materials.

## 1. Introduction

Cement-based materials are a cornerstone of modern construction practices, valued for their versatility and enhanced performances. They are widely applied across a variety of settings, ranging from large-scale infrastructure projects to specialized construction techniques [1,2]. A critical factor influencing their successful application is their rheological behaviors. Rheology, which governs the flow and deformation of fresh concrete, plays key roles in determining the workability, stability, and overall performance [3,4].

In self-compacting concrete (SCC), for instance, the ability to flow under its own weight while resisting segregation and ensuring full compaction is directly related to its rheological properties [5,6]. In 3D-printing concrete (3DPC), precise control over the rheology is essential for achieving stable, layer-by-layer deposition without collapse or deformation [7,8]. Shotcrete, applied at high velocity, also requires a balance between pumpability and rapid setting to ensure proper adhesion and buildability [9,10]. The flow behavior of concrete affects not only placement ease but also performance attributes, such as strength, durability, and cracking resistance. Key rheological parameters, such as yield stress, plastic viscosity, and thixotropy, significantly influence workability, segregation resistance, and pumpability, all of which are critical for successful construction outcomes.

In recent years, a wide range of materials—including supplementary cementitious materials (SCMs), chemical admixtures, and nanomaterials—have been extensively used to modulate the rheological properties of cement-based systems. Among these, SCMs, such as fly ash (FA), ground granulated blast furnace slag (GGBFS), and silica fume (SF), have attracted significant attention. These materials not only help to reduce the cement content—thereby lowering the carbon emissions associated with cement production—but also influence the rheology of cementitious systems. Given the environmental challenges of cement production, integrating SCMs offers a sustainable way to reduce cement consumption while improving concrete performance. The unique particle shapes, sizes, and chemical compositions of SCMs play crucial roles in adjusting properties such as yield stress, plastic viscosity, and thixotropy, thereby improving workability, enhancing durability, and mitigating segregation [11,12,13,14].

In addition to SCMs, chemical admixtures, like superplasticizers (SPs), viscosity-modifying agents (VMAs), setting-time control agents, and superabsorbent polymers (SAPs) are essential for optimizing the rheological properties of cement-based materials. These additives enable precise control over factors such as flowability, cohesiveness, and segregation resistance, making them particularly valuable in demanding construction applications. VMAs improve stability by modifying viscosity and preventing segregation, while setting-time control agents regulate the hydration process to adjust setting times for different construction needs. SAPs, with their ability to absorb and retain water, improve workability and help to mitigate early-age shrinkage. Furthermore, the use of phase change materials (PCMs) [13] in cement paste might significantly affect the rheological properties, including the viscosity, yield stress, and temperature-sensitive behavior, of the paste. Solid PCMs reduce workability and enhance the thixotropic behaviors of cementitious materials, while liquid PCMs can temporarily lower viscosity but may cause segregation [14]. The challenges of using PCMs include sedimentation, inconsistent setting times, encapsulation damage, interference with hydration, increased water demand, and long-term durability concerns.

Nanomaterials, such as nano-silica (NS), nano-clays (NCs), carbon nanofibers (CNFs), and graphene oxide (GO), further refine the rheological performances of cement-based materials because of their large surface areas and reactivities. These materials enhance concrete’s stability, flow behavior, and mechanical properties, underscoring their growing importance in advanced concrete formulations. Understanding the interactions between nanomaterials and the cement matrix is the key to fully exploiting their potential, and this review aims to explore these interactions.

Through the strategic integration of these materials, it is possible to tailor the rheology of cement-based materials to meet the specific demands of various construction techniques while addressing sustainability concerns associated with high cement usage. Existing reviews, such as those by Khayat et al. [15], have focused primarily on the rheology of ultrahigh-performance concrete (UHPC), limiting a comprehensive understanding of cement-based material systems as a whole. Similarly, Vijayan et al. [16] reviewed specific materials, like nano-silica, but did not provide an in-depth analysis of the broad spectrum of additives and their synergistic effects. Some reviews have only discussed the influences of individual admixture types on the rheological properties of cement-based materials [1,17,18]. Up to now, there still lacks a comprehensive and systematic framework that classifies additives based on their material properties and analyzes their effects on the rheological performances of cement-based materials.

This review is aimed at researchers and engineers in the field of cement-based materials, particularly focusing on the optimizing of the performance of fresh cement-based systems for advanced applications, such as 3D-printed concrete and shotcrete. This study uses a systematic methodology to provide a comprehensive understanding the impacts of additives on the rheological properties of cement-based materials. The relevant literature was identified through extensive searches in Scopus, Web of Science, and Google Scholar databases. Publications were carefully screened to include peer-reviewed articles, high-impact journals, and key review papers from the last several decades. By synthesizing the findings from these sources, this review systematically classifies various additives based on their distinct material properties and provides an in-depth analysis of their effects on the rheological performances of cement-based materials, particularly focusing on their roles in regulating performances in both the fresh and hardened states. By comprehensively summarizing the rheological effects of different categories of additives, this paper reveals the specific contributions of each type of additive in optimizing rheological properties. Compared to the existing literature, this review offers a more systematic and comprehensive approach in material selection and classification, presenting a new theoretical framework for optimizing the rheological performances of cement-based materials. Through a detailed analysis of the types of additives and their rheological mechanisms, this review offers a fresh perspective for optimizing the performances of cement-based materials, advancing the application of cement-based materials in the construction field.

## 2. Supplementary Cementitious Materials

Supplementary cementitious materials (SCMs) are commonly used as additives in concrete mixtures. The cement industry is a significant contributor to global CO_2_ emissions. Thus, SCMs are increasingly employed as substitutes for ordinary Portland cement to mitigate the environmental impacts of concrete production. By carefully selecting and adjusting the type and dosage of SCMs, it is possible to enhance specific properties of concrete, particularly its rheological and mechanical performances. This section will explore the influences of SCMs on the rheological properties of concrete, elucidating their roles in improving mixture workability, reducing water demand, and contributing to the sustainability of the construction industry.

### 2.1. Fly Ash

Fly ash (FA) is an industrial byproduct collected from coal-fired power plants, where finely ground coal is burned. Composed primarily of reactive SiO_2_ and Al_2_O_3_, FA exhibits pozzolanic properties, which allow it to react with Ca(OH)_2_ during the cement hydration process to form additional calcium silicate hydrate (C-S-H) and calcium aluminate hydrate (C-A-H). These reactions densify the matrix, leading to improved strength and durability. Additionally, the incorporation of FA into concrete significantly reduces the heat of hydration—because of the exothermic nature of cement hydration and the low thermal conductivity of concrete—thereby mitigating the risk of cracking [19]. Fly ash exists in two main variations: class-C fly ash (C-FA) and class-F fly ash (F-FA), each with distinct chemical and mineralogical compositions. C-FA typically contains higher levels of calcium, which give it both pozzolanic and cementitious properties, making it more reactive and capable of contributing to the early strength development of concrete. F-FA, on the other hand, has a lower calcium content and primarily exhibits pozzolanic activity, contributing to the long-term strength and durability of concrete. Research has shown that class-C fly ash reduces the slump and entrained air content of fresh concrete because of its higher CaO content, while class-F fly ash does not significantly affect these properties, maintaining similar workability to that of the control mixture [20]. These differences in composition play significant roles in determining how each type of fly ash affects the rheology, strength, and durability of concrete.

**Figure 1:**
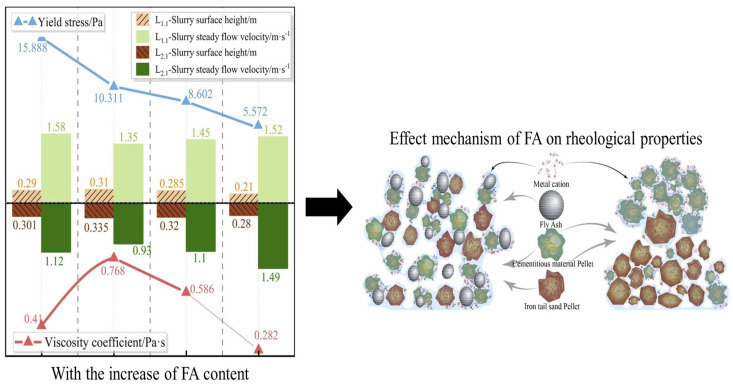
Effects of FA on the rheological parameters of cement-based materials and its mechanism [23].

Incorporating FA into cementitious materials influences their rheological behaviors, especially when used as a partial replacement for cement. Thiyagarajan et al. [21] investigated the effects of incorporating 20–80% F-FA into cement and concrete. Their findings indicated that with increasing F-FA content, the yield stress and viscosity of cementitious materials decrease, attributed to the ball-bearing effect of the F-FA particles. This results in enhanced rheological performance. Further research by Luo et al. [22] explored the rheological properties of fresh pastes containing FA by measuring and calculating parameters such as the water film thickness, yield stress, apparent viscosity, and thixotropy. The results indicated that increasing the FA content decreases the water film thickness, yield stress, apparent viscosity, and thixotropy of the fresh paste. Studies have also shown that fly ash can effectively mitigate segregation in cement mortar. Specifically, at an FA content of 8–12%, the yield stress of the mortar was reduced by 35.1–64.9% [23]. The use of ultrafine FA, because of its smaller particle size, can fill the voids in cementitious materials more effectively and further enhance the ball-bearing effect, thereby significantly improving the rheological properties [24]. The incorporation of F-FA into cementitious materials can enhance their rheological performances to a certain extent (see Figure 1). By adjusting the particle size and dosage of the FA, its rheological properties can be tailored to meet specific material requirements [25].

As a further development of FA, fly ash cenosphere (FAC) is a specialized form of FA, characterized by its hollow spherical structure, low density (typically 400–800 kg/m^3^), and partial reactivity [26]. Its lightweight nature and beneficial properties make it a popular choice as a filler in various concrete applications. To optimize the use of FAC in concrete, its effects on rheological properties are essential.

FAC exhibits a “ball-bearing” effect that enhances the performance of SCC mixtures in the fresh state. Mixtures containing FAC (at 5% of the cementitious material’s weight) demonstrate higher static yield stress, higher dynamic yield stresses, and lower viscosities [27]. Lu et al. [28] explored the incorporation of FAC and an air-entraining agent (AEA) into 3D-printing cementitious materials, aiming to achieve an optimal mixture design that meets the specific delivery and deposition requirements of 3D printing. Rheological tests revealed that a mixture with a 100% FAC substitution and 0.1 g/L AEA (designated as M-100%-0.1) exhibited the lowest dynamic yield stress and plastic viscosity while maintaining a sufficiently high static yield stress. Given these properties, they concluded that the M-100%-0.1 mixture is particularly suitable for spray-based 3D-printing techniques. Further investigations by Lu et al. [29] assessed the impacts of adding MgO and FAC on the setting, hydration, and rheological properties of fresh mixtures. The findings were consistent with those of their earlier study, demonstrating that FAC introduction reduces the dynamic yield stress and plastic viscosity, because of its spherical shape, while increasing the static yield stress. Moreover, they found that mixtures with 20 wt.%/40 wt.% FAC additions experienced a reduction in pumping pressure by 29%/31% and an increase in the critical ratio by 78%/68% compared to those of plain MgO-activated slag material. These results suggest that a material with a tailored rheology enhances the delivery and deposition performance of the mix, ultimately facilitating superior 3D-printing quality.

In conclusion, FAC is a highly effective additive for enhancing the rheological performances of cementitious materials. Its lightweight, spherical structure contributes to improved flow properties and optimized yield stress, making it particularly beneficial in applications such as SCC. FAC improves workability by enhancing both flowability and stability, allowing for better handling of the mixture. These attributes position FAC as a valuable component in modern concrete formulations, contributing to the development of high-performance and sustainable construction materials.

### 2.2. Ground Granulated Blast Furnace Slag

Ground granulated blast furnace slag (GGBFS) is a byproduct generated during the production of pig iron in a blast furnace and is primarily composed of silicates, calcium, and alkaline silicates containing aluminum. It exists in the form of fine, non-crystalline, glassy particles. When finely ground, GGBFS can be used as a supplementary cementitious material, either alone or in combination with other materials, to significantly enhance the performance of concrete [30]. However, the rheological effects of GGBFS are not universally positive and depend critically on the chemical compatibility between the slag and cement phases, particularly the tricalcium aluminate (C_3_A) content in the cement [31]. The replacement of cement with GGBFS improves the workability of cementitious materials [32]. For example, Maameri et al. found that the incorporation of 15% and 25% GGBFS alleviated the reduction in the rheological performances of cement-based materials [33]. Nevertheless, recent studies have highlighted that excessive GGBFS substitution in high-C_3_A cements may lead to accelerated sulfate–aluminate reactions, resulting in increased yield stress and reduced flowability because of premature ettringite formation [34,35]. The improved dispersibility and surface characteristics of GGBFS particles enhance the workability of concrete, as the smoother and denser slag particles absorb less water during mixing [30]. Research by Park et al. demonstrated that the yield stress of an ordinary Portland cement (OPC)-GGBFS cementitious system initially decreases and then increases with the increasing addition of GGBFS, reaching its lowest value at a GGBFS content of 30%. However, the plastic viscosity decreases consistently as the GGBFS content increases [36].

**Figure 2:**
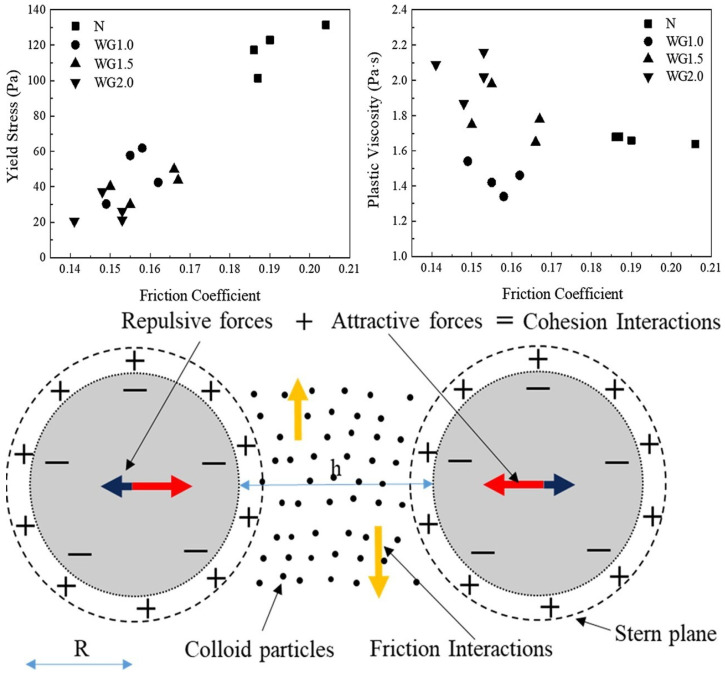
The rheological mechanism of the AAS paste [38].

In contrast to GGBFS as a supplementary cementitious materials, alkali-activated slag (AAS) constitutes an entirely distinct binder system. Alkali-activated slag concrete is produced through the chemical activation of GGBFS by alkaline solutions (e.g., sodium silicate or sodium hydroxide), forming a calcium aluminosilicate hydrate (C-A-S-H) gel rather than the conventional calcium silicate hydrate (C-S-H) of Portland cement. These fundamental differences in the reaction mechanisms and binder chemistry explains why AAS research receives focused attention: it represents a paradigm shift toward low-carbon alternatives to traditional cement, with unique rheological challenges requiring specialized optimizations (e.g., activator type, slag fineness, and curing conditions). Sun et al. investigated the effects of mixing conditions and the type of activator anions on the rheology of alkali-activated slag concrete. They found that extending the mixing time from 3 to 5 min reduced the static and dynamic yield stress of the concrete by 25% and 23%, respectively, because of the breakdown of solid particles and agglomerates formed in the early stages. Additionally, replacing the sodium silicate activator with an equivalent amount of a sodium carbonate activator significantly improved the dynamic flowability of the concrete while increasing its static yield stress and thixotropy [37]. Tian et al. [38] explored the rheological mechanism of AAS paste by calculating the parameters, such as the zeta potential, surface energy, hydrogen bonding, and friction coefficient, of the alkaline activators (as shown in Figure 2). Their findings indicated that the yield stress of the AAS paste is primarily determined by frictional interactions between particles, while plastic viscosity is mainly influenced by attractive interactions, including van der Waals forces and Lewis acid–base interactions.

These studies highlight the crucial roles of GGBFS and its alkali-activated forms in optimizing the rheological properties of cementitious materials. By effectively controlling the yield stress, plastic viscosity, and thixotropy, GGBFS enhances concrete’s workability and stability, supporting the development of more sustainable and efficient concrete technologies.

### 2.3. Silica Fume

Silica fume (SF), also known as micro-silica, is a very fine powdery material composed primarily of amorphous silicon dioxide (SiO_2_). It is a byproduct generated during the production of silicon metal or silicon alloys. Because of its high fineness degree and large specific surface area, SF is widely used as a supplementary cementitious material to significantly enhance the performance of cement-based products.

**Figure 3:**
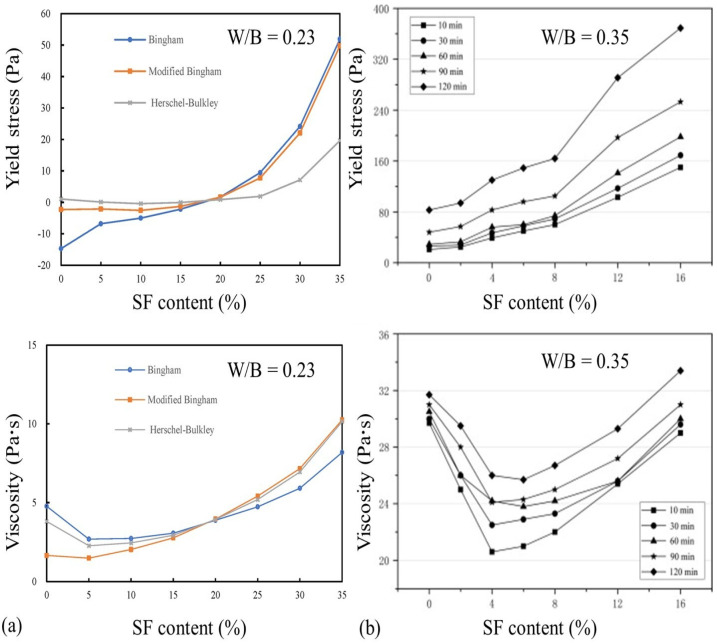
Effects of different SF contents on the rheological properties of cement-based materials: (**a**) W/B = 0.23 [40]; (**b**) W/B = 0.35 [41].

SF can be utilized to modify the rheological properties of cementitious materials [15], see Figure 3. Smirnov et al. [39] investigated the effect of SF on the rheology of self-compacting mortar, revealing that the large specific surface area of SF particles requires an increased water-to-binder ratio (up to 30%) to achieve similar workability when replacing cement with SF. For fresh cementitious materials, when the SF content is 15% or less, changes in its dosage do not significantly affect the yield stress, and the paste exhibits characteristics of a linear Newtonian fluid. However, when the SF content exceeds 15%, the yield stress increases sharply with increasing SF content, while the plastic viscosity and the area of the hysteresis loop initially decrease and then increase, indicating shear-thinning behavior typical of a pseudoplastic fluid [40].

Lu et al. [41] further explored the impacts of SF on the rheological properties, showing that when the SF content was less than or equal to 8%, its addition had a limited effect on the paste’s slump flow value (TW). However, when the SF content was 12% or higher, the addition of the SF significantly reduced the TW. The yield stress of the paste increases with increasing SF content, and the rate of the increase is moderate at lower TW values but accelerates at higher TW values. Moreover, with increasing SF content, the plastic viscosity of the paste initially decreases and then gradually recovers.

These findings highlight the critical role of silica fume in fine-tuning the rheological behavior of cementitious materials, where its optimal dosage can significantly influence the workability, yield stress, and viscosity of the mix. Understanding these effects allows for more precise control of concrete mixtures, particularly in applications requiring specific flow characteristics, such as self-compaction or 3DPC. As such, careful consideration of the SF content is essential in achieving the desired balance between workability and mechanical performance in advanced cementitious systems.

In addition to the conventional mineral admixtures mentioned above, the impacts of metakaolin on the rheological properties of concrete have also been studied. Barkat et al. [42] discovered that the incorporation of metakaolin into self-compacting limestone cement concrete mixes not only reduced the heat evolution but also moderated the yield stress and increased both the plastic viscosity and V-funnel flow time. These changes contributed to improved rheological properties, such as flowability, passing ability, and segregation resistance. In contrast to the findings of Barkat et al. [42], research by Rojo-López et al. [43] indicates that metakaolin can increase the rheological parameters of self-compacting concrete mixtures, including plastic viscosity and dynamic yield stress, which aids in regulating its rheological performance. This discrepancy may be because of the differences in the systems studied and the dosage of the metakaolin used. Furthermore, metakaolin can improve the workability of concrete, and its effectiveness surpasses that of SF [44].

In summary, the use of SCMs, such as FA, GGBFS, SF, and others, plays a crucial role in modifying the rheological properties of concrete. Each type of SCM impacts key rheological parameters, like yield stress, plastic viscosity, and thixotropy, in distinct ways, allowing for precise adjustments to enhance workability and performance. Table 1 summarizes the effects of these SCMs, including metakaolin (MK), coral powder, limestone fines (LFs), rice husk ash (RHA), and so on, which are increasingly considered for sustainable concrete formulations.

MK is a highly reactive pozzolanic material derived from thermally activated kaolinite (600–900 °C). Its incorporation into cementitious systems significantly influences rheological properties through both physical and chemical mechanisms. The large specific surface area of MK increases the water demand, reducing the amount of free water available for lubrication. This leads to decreased flow spreads (up to a 48.8% reduction at 20% MK) and flow rates. Despite the reduced flowability, MK enhances the yield stress (a 234.2% increase) and plastic viscosity (a 661.3% increase) by promoting flocculation via electrostatic attraction and denser particle packing. In addition, MK-modified pastes exhibit pronounced thixotropic recovery because of enhanced interparticle cohesion. The dual role of MK as a filler and pozzolan allows for simultaneous rheological control and mechanical enhancement [45,46,47].

Coral powder enhances the yield stress and viscosity in cement-based materials, primarily because of its large specific surface area and unique chemical composition, which promote stronger particle interactions within cement pastes. These characteristics can enhance cohesion and reduce the bleeding of cement-based materials. Furthermore, the use of coral powder in concrete can contribute to the development of more sustainable and eco-friendly concrete, reducing the reliance on clinker. Rice husk ash, a byproduct of rice milling, also plays roles in increasing viscosity and modifying the rheological behavior of concrete, reducing segregation and improving stability. Its fine particles and pozzolanic properties help to increase viscosity, improving the uniformity and cohesiveness of the mixture.

Limestone powder, as a widely utilized SCM, plays a pivotal role in tailoring the rheological properties of cement-based systems. Its effects stem from a combination of physical, chemical, and microstructural mechanisms, which have been extensively explored in recent studies.

The incorporation of limestone powder significantly influences rheological parameters, including yield stress, plastic viscosity, and thixotropy, through multiple pathways. First, its particle morphology—characterized by rounded or sub-angular shapes—facilitates a “bearing effect” that reduces interparticle friction, thereby enhancing flowability and lowering yield stress [48,49]. Second, limestone contributes to dissolution–precipitation processes during hydration. Although limestone itself is chemically less reactive than clinker, its partial dissolution releases Ca^2+^ ions, which interact with aluminates to form carbo-aluminate phases. These reactions not only refine the pore structure but also modulate the kinetics of the hydration, indirectly affecting the rheological evolution over time [50]. For example, Muzenda et al. [48] observed that limestone’s dissolution–precipitation behavior enhances structural rebuilding during resting periods, subtly increasing the static yield stress while maintaining a lower dynamic yield stress. Furthermore, the role of limestone in modulating harmonic distortion—a measure of the nonlinear viscoelastic response—has been highlighted in advanced rheological studies. Under large-amplitude oscillatory shear, limestone-rich pastes exhibit higher harmonic distortion, indicative of viscous liquid behavior [51].

Additionally, limestone filler enhances thixotropy, aiding in structural build-up during rest, making it ideal for applications like self-compacting concrete and 3D printing. Limestone filler significantly influences the rheological properties of cement-based materials by enhancing flowability and stability through improved particle packing and reduced water demand. It lowers the plastic viscosity and yield stress because of its dilution effect while also acting as a nucleation site for hydration, promoting cohesiveness and accelerating reactions [52].

However, the dosage of the limestone must be carefully optimized. Excessive limestone can dilute the clinker content, slowing early hydration and reducing the mechanical strength. Recent studies have also emphasized the importance of the water film thickness (WFT) in limestone-modified systems. Large-specific-surface-area limestone (e.g., ultrafine grades) reduces WFT, increasing interparticle attraction and viscosity, whereas coarser limestone enhances WFT, improving dispersion [53].

These materials, when integrated with other admixtures, offer significant potential for optimizing concrete formulations, especially for advanced construction applications, where specific rheological characteristics are essential. When combined with other admixtures, these materials significantly enhance the potential for optimizing concrete formulations, making them particularly valuable for advanced construction applications, where specific rheological characteristics are essential.

**Table 1:** Rheological properties and dosages of supplementary cementitious materials.

Type	Dosage (%)	Plastic Viscosity (mPa·s)	Yield Stress (Pa)	Thixotropy (Δ)	Remarks	Reference
FA	5–25	As FA substitution increases, the shear-thickening effect and slurry viscosity grow.	Yield stress demonstrates a proportional relationship with the FA content.	-	The large specific surface area of SF increases water wetting, leading to larger flocculated structures, higher yield stress, and poor fluidity in pastes.	[54]
	8–12	The plastic viscosity of the mortar was 0.768 Pa·s by adding 8% fly ash.	Adding 8–12% FA reduced the yield stress of the mortar by 35–65%.	-	FA effectively improved mortar segregation and precipitation.	[23]
	10 and 20	Slurries mixed with 10% and 20% FA increased the early plastic viscosity by 0.11 and 0.17 Pa·s, respectively, compared to that of the control cement-based material without FA.	After 57 min of hydration, the dynamic yield stresses were 75.1, 87.0, 190.8, and 132.0 Pa. The Paste with 20% FA consistently exhibited the highest dynamic yield stresses.	Using 20% FA reduced the static yield stress and thixotropic by 31.9 Pa and 971.1 Pa·s^−1^. The appropriate FA content reduced the yield stress and enhanced the thixotropy.	FA increased the plastic viscosity of the cement-based material by promoting early hydration and consuming more free water.	[55]
GGBFS	0–50	As the GGBFS content increased from 0% to 50%, the plastic viscosity increased from 7.13 Pa·s to 16.46 Pa·s.	As the GGBFS content increased from 0% to 50%, the yield stress of the cement-based material increased from 36.6 Pa to 239.1 Pa.	As the GGBFS amount increased, the thixotropy of the cement-based materials increased over the resting time.	Low GGBFS contents (≤30%) mainly affected the plastic viscosity of the geopolymer, while high GGBFS contents (40–50%) had a remarkable effect on the yield stress.	[56]
	10–40	An increase in the slag content led to a decrease in the plastic viscosity compared to that of the control mortar.	Using slag reduced the yield stress of the paste, with reductions of 10% to 55% for pastes with 10% to 35% slag.	-	The increase in the slag content increased the yield stress and plastic viscosity of fresh mortars.	[57]
SF	0–35	The plastic viscosity and hysteresis loop area decreased slightly with the addition of a small amount of SF but increased significantly with the continuous increase in SF.	When the SF content was less than 15%, the yield stress was close to 0. With increasing SF content, the yield stress increased rapidly.	-	As the SF content increased from 0% to 35%, the shear-thickening effect of the cement-based materials decreased, transitioning from dilatant to Newtonian and then to pseudoplastic behaviors.	[40]
	0–50	Adding SF increased the plastic viscosity by 6.7–24.7%. The nucleation and filling of the SF promoted the hydration reaction and significantly increased the plastic viscosity of the paste.	The SF addition increased the yield stress by 2.6–18.4%, with its small particle size enhancing nucleation sites and accelerating hydration.	-	The SF increased the rheological parameters and reduced the fluidity attenuation, with a stronger effect during accelerated hydration, enhancing the reaction through nucleation and releasing more heat.	[58]
SF and FA	0–20	SF and FA significantly reduced the plastic viscosity of the cement-based materials, with reductions of 110% and 175%, respectively.	The yield stress decreased to 57 Pa with 10% SF then increased as the SF content rose further. The yield stresses of the pastes with 10% and 20% FA decreased by 104% and 315% compared to that of the reference.	-	SF and FA significantly improved the fluidity of cement-based materials.	[59]
Metakaolin (MK)	5–20	As the MK replacement increased from 5% to 20%, the plastic viscosity increased in the range 1.2–661.3%. The incorporation of MK can increase the plastic viscosity.	As the MK replacement increased from 5% to 20%, the yield stress increased in the range 5.1–234.2%. The incorporation of MK can increase the yield stress.	As the MK replacement increased from 5% to 20%, the thixotropy increased in the range 1.8–146.69%. The incorporation of MK can increase the thixotropy.	Adding MK can reduce flow rate and flow spread. Using MK reduced the film thickness of the particles, explaining its effects on both microscopic rheological parameters and macroscopic flow properties.	[45]
	5 and 10	When MK is used in cement-based materials, if attractive forces dominate the system, flocculation occurs, leading to increases in rheological parameters. This effect has been observed in studies involving MK.	The static yield stresses of the 5% and 10% MK mixtures were 522 and 764 Pa, respectively, showing a content-dependent increase. MK’s absorbent laminar structure reduced the requirement of the free water and increased the flow resistance.	The thixotropic behaviors of mixtures are favored by MK.	The incorporation of MK increases the flow resistance of the cementitious mixes, requiring greater force to flow, with the effect being proportional to the additional content.	[60]
Coral Powder (CP)	0–15	With the addition of the coral powder, the plastic viscosity (μ) also increased from 0.21 to 0.74, a rise of 243.3%.	With the addition of the coral powder, the yield stress (τ_0_) gradually increased from 1.20 to 3.71 Pa, a rise of 208.1%.	-	The addition of CP increased the yield stress and plastic viscosity of the paste, which affects the rheological properties of pastes.	[61]
MK and CP	10 CP 5–30 MK	The paste with 10% CP showed higher consistency and plastic viscosity. The reduction in the rheological index indicated pronounced shear thinning, except when adding 30% MK.	The cement paste with 10% CP showed higher dynamic shear stress, which was further increased by adding MK, indicating greater yield stress.	-	The ternary system showed significant shear thinning, with the Herschel–Bulkley model fitting well. Up to 20% MK enhanced the rheology and workability.	[62]
Limestone Fines (LFs)	0–60	-	The rheological properties of the paste made with LFs showed viscoplastic fluids with shear thinning behavior.	-	Incorporating LFs into cement suspensions increased the yield stress, viscosity, and flow time, reducing the fluidity.	[51]
	30, 60 vol.	Using 30% LF1 increased the torque of the mortar at higher speeds, resulting in a 16% increase in the plastic viscosity. At 60% replacement by LF2, the plastic viscosity was reduced by 8%.	Using 30% LF1 did not affect the yield stress. Adding LF2 reduced the yield stress, with an 11% decrease at a low rpm for the composition; 60% replacement decreased the yield stress by 18%.	-	Limestone type and the use of a dispersant affected the rheological parameter yield torque.	[52]
LFs and Rice Husk Ash (RHA)	10 RHA 5–20 LFs	When 10% RHA was adopted, the consistency (plastic viscosity) decreased by 28.17%, 40.85%, and 73.24% compared to those of the cement pastes without LFs.	With 10% RHA, adding 5%, 10%, and 20% LFs reduced the cement paste’s dynamic yield stress by 43.86%, 69.64%, and 87.11%, respectively.	LFs and RHA have the opposite effects on the static yield stress and structural build-up rate. RHA boosts the initial static yield stress and accelerates structural recovery.	LFs effectively eliminated the adverse effect of the RHA on the rheological properties. The pastes with a combination of 10% RHA and 10% or 20% LFs had had suitable rheological properties.	[63]

## 3. Chemical Admixtures

In addition to SCMs, chemical admixtures play a vital role in enhancing the rheological properties of cementitious mixtures [3]. Research has demonstrated that the rheology of these materials is significantly influenced by factors such as the type and content of fillers, the water-to-cement (w/c) ratio, and the dosage of superplasticizers (SPs) and by parameters like ultrasonic treatment times and mixing rates. Among these factors, the large specific surface areas of fillers have been identified as a key determinant of cement pastes’ rheology [64]. Moreover, various chemical admixtures, including viscosity-modifying agents (VMAs), setting-time control agents, and superabsorbent polymers (SAPs), are increasingly used to optimize cementitious materials’ performance. VMAs enhance stability by modifying viscosity and preventing segregation, while setting-time control agents regulate the hydration process to adjust the setting time for different construction needs. SAPs, with their ability to absorb and retain water, improve workability and help to mitigate early-age shrinkage. The following sections will explore how these and other chemical admixtures contribute to controlling and optimizing the rheological behaviors of cementitious materials.

### 3.1. Superplasticizers

**Figure 4:**
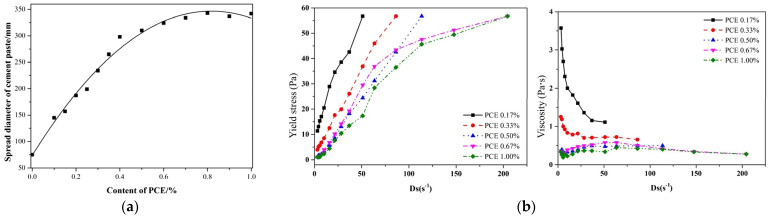
(**a**) The fluidity of the cement paste as a function of the PCE content; (**b**) The rheological performances of the cement paste as functions of the PCE content [68].

To enhance the rheological properties of cementitious materials, superplasticizers (SPs), which are chemical additives that significantly improve the workability of concrete, are widely used. SPs are typically classified into several types, including polycarboxylate-based superplasticizers (PCEs), naphthalene-based superplasticizers (NSPs) [65], and melamine-based superplasticizers (MSPs) [66], each with distinct molecular mechanisms that directly govern their effects on rheological parameters, such as yield stress, plastic viscosity, and thixotropy. Among these, PCEs, which are a commonly used type because of their superior performances, are known to significantly reduce the apparent viscosity and yield stress of fresh cement pastes. The carboxylate (–COO^−^) groups in PCEs adsorb onto positively charged cement particles (e.g., C_3_A phases) via electrostatic interactions, while the polyethylene oxide side chains provide steric hindrance to prevent particle flocculation (see Figure 4) [67]. This dual mechanism (electrostatic + steric) efficiently disperses cement agglomerates, releasing entrapped water and reducing both the yield stress and plastic viscosity [68].

However, the effectiveness of SPs can vary depending on the interaction between the superplasticizer and the type of cement used. The cement composition critically modulates the SP’s performance. NSPs and MSPs, classified as sulfonated polymers, primarily rely on electrostatic repulsion from sulfonate (–SO_3_^−^) groups adsorbed on cement surfaces [69]. Although effective in reducing yield stress, their linear polymer chains lack long side groups for steric stabilization, resulting in higher plastic viscosities compared to those of PCEs. Additionally, NSPs and MSPs exhibit poor compatibility with cements containing high alkali contents because of competitive adsorption between SPs and sulfate ions.

In addition, Pott et al. [70] studied the incompatibility of particular SPs with OPC and observed that high dosages of SPs led to the rapid stiffening of the cement paste. Although rapid stiffening is typically undesirable in many traditional concrete applications, under certain conditions, such as when quick setting is required, this property can be beneficial. Thus, a thorough understanding of the optimal type and dosage of superplasticizers, along with their interactions with other components in cementitious mixtures, is crucial for customizing rheological properties to meet the varied performance demands across different construction applications.

In addition to traditional PCEs, recent research has explored several novel superplasticizers with promising results. Composite SPs, like those combining naphthalene- and melamine-based superplasticizers, significantly improve the workability and strength of cementitious materials by reducing plastic viscosity and enhancing fluidity retention through their synergistic effects [71]. Stecher et al. [72] synthesized an innovative class of polyphosphate superplasticizers, which demonstrated superior dispersing capabilities when compared to those of conventional PCEs. This was achieved through a copolymerization reaction involving 2-(methacryloyloxy) ethyl phosphate monomer and polyethylene glycol methacrylate ester at varied molar ratios [72]. These novel superplasticizers not only exhibited less retardation of cement hydration but also enhanced adhesion to the cement surface, showcasing a significantly high calcium-binding capacity. Additionally, a new type of polycarboxylate superplasticizer, termed nano-micelles (nano-PCEs), was developed by Chen et al. [73]. These were prepared using aqueous emulsion copolymerization to create a core–shell structure. Experimental validation revealed that cement pastes incorporating these nano-PCEs maintained better fluidity for up to three hours. Furthermore, Chen et al. [74] designed a new type of PCE superplasticizer by incorporating different hydrophobic ester groups and studying their effects on the rheological properties and flow behaviors of mortars. Their findings revealed that the hydrophobic ester groups enhance the adsorption capabilities of PCEs and increase the electrostatic repulsion between cement particles, thereby releasing a greater amount of free water within the mix. This, in turn, significantly improves the dispersion performance and rheological properties, such as the shear stress and apparent viscosity.

Considering the occasional incompatibilities between cement types and SPs, the introduction of these advanced SPs generally improves the flowability of the cement paste. This enhancement is particularly beneficial in applications that demand superior rheological properties, where precise control over the material behavior is crucial, such as in high-performance building constructions and complex structural applications.

### 3.2. Viscosity-Modifying Agents

VMAs are pivotal chemical admixtures used to enhance the rheological properties of cement paste. These agents, which include acrylic polymers and polysaccharide-based biopolymers derived from cellulose, starch, or bacterial fermentation, are essential in improving the robustness of highly fluid cementitious materials against bleeding and segregation [75,76,77]. The mechanisms of action of VMA molecules on cement particles are categorized into water retention, polymer–polymer entanglement, and adsorption with particle–particle bridging. Typically, the adsorption of VMAs leads to poor dispersion of cement particles, manifesting as thixotropic behavior, which, consequently, increases the yield stress and plastic viscosity of cement-based materials [3].

Studies in the past decade have laid the groundwork for the use of biologically derived VMAs in cementitious materials. For example, Azima et al. [78] explored the impact of incorporating *Sporosarcina pasteurii* (*S. pasteurii*) cells, a novel biological VMA, into cement paste on the paste’s properties. Their findings indicated that these cells significantly increased the plastic viscosity and yield stress of the paste. These enhancements were attributed to the presence of polysaccharides and peptidoglycans in the cell structures, which contribute to higher water retention. Pei et al. [79] also utilized the bacterial cell walls (BCWs) of *Bacillus subtilis* as biological VMAs to modulate the performance of cement pastes. The use of BCWs not only increased the apparent viscosity and yield values of the cement pastes but also significantly improved their resistances to bleeding and segregation. These earlier studies highlight the ongoing interest in and the evolving nature of biologically derived VMAs in construction materials. Building on these foundational studies, Álvarez et al. [80] analyzed the impacts of bio-based viscosity-modifying admixtures (BVMAs), under various dispersion conditions, on the rheological properties of cement pastes. Their results indicated that the BVMAs increased the viscosity of the cement pastes.

VMAs primarily function by adsorbing onto the surfaces of adjacent cement particles and introducing bridging forces within the system, thereby increasing the yield stresses of cement-based materials. However, the combined use of VMAs and SPs can lead to some incompatibilities in the cement pore solution, potentially causing competitive adsorption at particle surface positions, which affects the performances of cement-based materials [81]. Castellanos et al. [82] explored the effects of six types of VMAs on the rheological and thixotropic behaviors of concrete. They assessed how the content of SPs influenced the cohesive and thixotropic effects of VMAs. Their findings revealed that a lower SP content (1.55%) allowed for a more accurate analysis of the lag area and rheological parameters associated with VMAs. Moreover, among the six VMAs examined, those based on silica (SC and HS) and modified bentonite (F2) demonstrated the best rheological performances.

**Figure 5:**
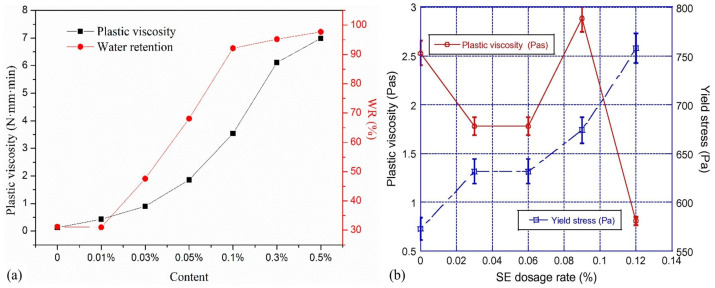
Effects of different VMAs on the rheological properties of cement-based materials: (**a**) HPMC [83]; (**b**) SE [84].

Further investigations by Chen et al. [83] explored the relationship between the plastic viscosity and water retention of cement mortar enhanced with hydroxypropyl methyl cellulose ether (HPMC). The findings depicted a linear positive correlation between the plastic viscosity and the water-retaining property, demonstrating that increased content of hydroxypropyl methyl cellulose ether leads to a corresponding rise in the water-holding capacity because of the elevated plastic viscosity. Additionally, Cappellari et al. [84] assessed the impacts of various polymer-thickening admixtures on cement mortars, including cellulose ethers of different molecular weights, guar ether, and starch ether (SE). The study found that the effects of these admixtures on the properties of fresh mortars could be categorized into two distinct groups: one, like starch ether, primarily influenced cohesion (the yield stress and storage modulus) rather than viscous dissipation (or the loss modulus); the other, comprising three types of cellulose ethers and guar ether, markedly enhanced the consistency of the mortar and resulted in a reduction in the apparent yield stress at high admixture concentrations. The effects of different VMAs on the rheological properties of cement-based materials are shown in Figure 5.

The evolving properties of VMAs offer significant opportunities for modern applications, such as 3D printing. Research conducted by Zhang et al. [8] has demonstrated the effectiveness of actively controlling the rheology of 3DPC through the coupling of temperature and viscosity modifiers to enhance printability. By optimizing the rheological properties of cement pastes, VMAs facilitate the practical implementation of 3DPC, thus accelerating its adoption and refinement within the construction industry. This highlights the imperative for ongoing research and development in VMA technology to fully exploit its potential in specialized applications.

In conclusion, the diverse functionalities of VMAs make them indispensable in the modern construction industry, particularly in enhancing the rheological properties of materials. Their abilities to improve water retention, reduce segregation, and increase yield stress and plastic viscosity address critical challenges in concrete applications.

### 3.3. Setting-Time Control Agents

**Figure 6:**
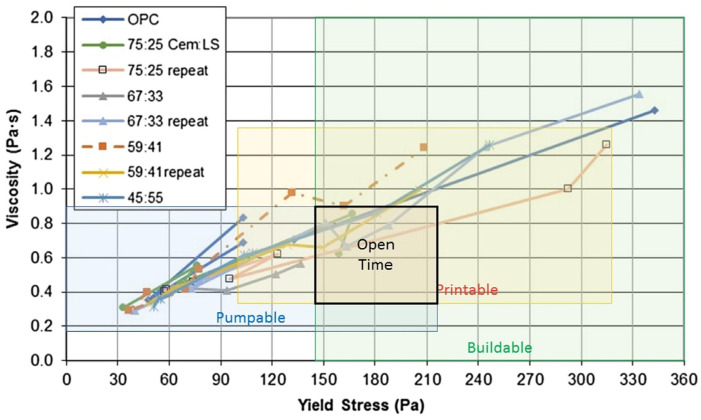
The viscosity and yield stress data for a set of cement pastes [85].

Given that setting times and rheological properties are critical parameters for the successful application of concrete in various construction methodologies, this section aims to review the effects of setting-time control agents on the rheology of concrete across different uses, specifically focusing on their application in 3D printing. The effective 3D printing of concrete necessitates operation within a defined open time window, which is characterized by the acceptable intersection of pumping, printing, and building capabilities (see Figure 6). In some cases, where certain combinations of materials and 3D printers are used, this intersection may not inherently exist. Therefore, understanding the precise timing and conditions under which optimal rheological properties are achieved is critical for the creation of structures that are both erect and durable [85]. To this end, the strategic use of setting-time control agents, such as retarders and accelerators, is considered to finely adjust the rheology of 3DPC.

Retarders are pivotal for moderating the rapid hardening of concrete. These agents offer several benefits, including delaying the peak of the hydration heat and enhancing workability [86,87,88]. The impacts of retarders on key rheological properties, such as yield stress and plastic viscosity, are explored in this section. Research has shown that the inclusion of retarders in the ettringite system of a cement slurry can extend the open time, thereby maintaining consistent flowability, as evidenced through rheological and hydration measurements [89]. By acting on the early hydration phases of 3DPC, retarders increase the mix’s fluidity, extend the setting time, and reduce both the yield stress and plastic viscosity, further enhancing the overall workability of 3DPC [90].

Chen et al. [91] investigated the adjustment of the initial setting and building times of sulfoaluminate cement (SAC) paste in 3D printing by incorporating boric acid (BA) and sodium gluconate (SG) as retarders. The results, illustrated in Figure 6, indicate that both BA and SG reduce the dynamic yield stress and plastic viscosity of the SAC paste, thereby enhancing both pumpability and printability.

The initial setting times of the SAC pastes with retarders showed a noticeable retardation effect post the addition of BA and SG, which also aids in controlling the building time. Similarly, Luo et al. [92] studied the effects of borax (BR) and SG as retarders on the properties of magnesium phosphate cement (MPC). The results demonstrated that the retarders BR and SG can improve the processability of MPC slurry, including lengthening the setting time and increasing fluidity. These improvements are because of the retarders slowing down the hydration reaction rate. Furthermore, the combined use of BR and SG has a synergistic effect on enhancing the performance of MPC mixtures.

In contrast to retarders, accelerators are employed to adjust rheological properties by increasing both the yield stress and plastic viscosity of fresh concrete. Pan et al. [10] documented that increases in the accelerator dosage correspondingly raise these properties. Moreover, Bentz et al. [85] highlighted the essential role of accelerators in ensuring a feasible and robust additive manufacturing process, as their addition can significantly bolster the yield stress. The encapsulation of accelerators for on-the-fly activation enables 3DPC to transition rapidly from a highly pumpable state to a hardened state with elevated yield strengths and moduli of elasticity. Compared to control printable mixtures, the activated concrete exhibited a fivefold increase in yield strength after activation [93]. Rubin et al. [94] produced three concrete mixtures with cohesive flow properties, using different ratios of accelerators to evaluate the improvements in yield strength gains. The results demonstrated that when using sulfate-based accelerators, the action of the acceleration leads to the formation of large amounts of ettringite, causing the yield stress of the concrete to increase exponentially, even within the initial few minutes.

Given that setting times and rheological properties are critical parameters for the successful application of concrete across various construction methodologies, this section reviews the effects of setting-time control agents on the rheology of concrete. Although specifically focusing on their application in 3D printing, the principles discussed herein are broadly applicable to other construction techniques that require precise control over concrete properties. Building on current research findings, it is evident that both retarders and accelerators influence the rheological properties of concrete to various extents. By adjusting the type and amount of these additives according to actual needs, it is possible to obtain the target parameter, ensuring the high-performance application of concrete.

### 3.4. Superabsorbent Polymers

SAPs act as a novel chemical admixture for cement-based materials, possessing unique water-absorbing and -releasing characteristics within the cement matrix. By modulating the hydration process of cement [95,96], SAPs significantly affect various properties of materials, such as strength and workability. Additionally, the impacts of SAPs on the rheological properties of pastes are noteworthy and warrant further investigation. Bessaies-Bey et al. [97] examined the influences of SAPs on the rheology of fresh pastes. Their research highlighted that SAP micro-gels can adsorb across multiple cement grains, effectively bridging them and thereby increasing the macroscopic yield stress of the suspension. Mechtcherine et al. [98] investigated the rheological behaviors of fresh-cement-based mortars modified with two types of SAPs, finding that the grading of SAPs significantly influences the development of the yield stress and plastic viscosity over time. Liu et al. [99] investigated the rheological changes in UHPC by incorporating SAPs. Their study revealed that the inclusion of SAPs significantly affected the yield stress and plastic viscosity of the UHPC over time. Notably, UHPC mortars containing SAPs exhibited substantial increases in yield stress because of prolonged water absorption. Additionally, different SAP formulations variably influenced the plastic viscosity and thixotropy of the mortars. Some SAPs reduced these properties, while others increased them, underscoring how UHPC’s rheological behavior is closely tied to the water absorption and desorption characteristics of the SAPs used. Kuruva et al. [100] studied the effects of SAPs on the rheological properties of SCC. The results indicated that the addition of dry SAPs altered the rheological performance of the SCC. The reduction in the amount of available water in the SCC matrix led to decreases in the yield stress and plastic viscosity. Additionally, the dry SAPs absorbed moisture from the mixture, which resulted in reduced workability.

Paiva et al. [101] observed that SAPs can function as water-retaining admixtures, though this reduces workability by removing water from the system, which increases torque and reduces fluidity. Pourjavadi et al. [102] examined the rheological properties of fresh pastes containing SAPs and colloidal silica nanoparticles, noting that increased quantities of these additives reduced yield stress and increased viscosity. They suggested that swollen SAP particles might lubricate the matrix or rapidly deswell in response to calcium carbonate precipitation, releasing absorbed water. Secrieru et al. [103] investigated the effects of SAPs in strain-hardening cement-based composites at temperatures ranging from 10 °C to 30 °C, finding that SAPs consistently increased the plastic viscosity and yield stress, irrespective of the desorption behavior in the cement pore solution.

**Figure 7:**
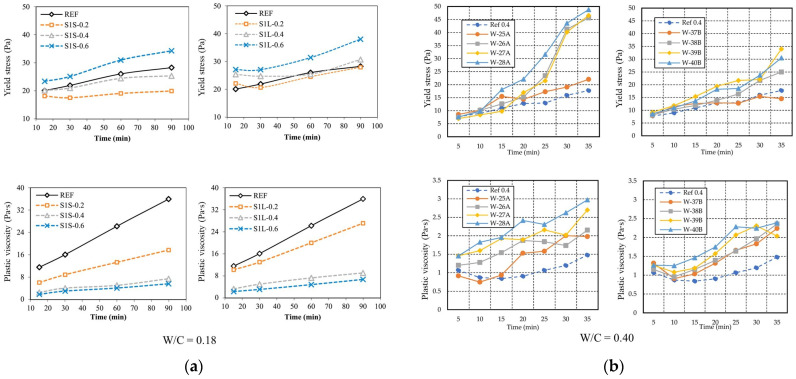
Rheological properties of cement-based materials with different W/C ratios and various SAP particle sizes and dosages (**a**) W/C = 0.18 [99]; (**b**) W/C = 0.40 [105].

Geng et al. [104] investigated how different particle sizes, contents of SAPs, and additional water affected the apparent viscosity of cement paste over time. They also assessed the correlations among the plastic viscosity, yield stress, and apparent viscosity. Their findings indicated that the particle size of SAPs significantly influences the apparent viscosity of the cement paste, with larger SAP particles showing more pronounced water absorption and desorption changes. Adding SAPs and extra water decreased the plastic viscosity and yield stress of the cement paste, thereby enhancing its flowability. Adsul et al. [105] analyzed the impacts of different-sized SAPs on the rheological properties of cement paste, revealing that smaller-sized SAPs have more significant effects on the plastic viscosity and yield stress, with the introduction of SAPs increasing both these properties (as illustrated in Figure 7). Dilbas [106] explored how the type of cement and the water-to-cement ratio affect the rheological properties of cement pastes modified with high-absorption polymers. Because of the SAPs’ absorption of moisture from the environment, there were increases in the viscosity, shear stress, and yield stress of the cement pastes. Increasing the water-to-cement ratio can mitigate some of these effects to an extent. Furthermore, as the fineness of the cement increases, the surface areas of cement particles also increase, leading to more effective absorption of environmental moisture by the cement particles, thus impacting the rheological properties of the cement paste.

There has been extensive research on the effects of SAPs as additives on the rheological properties of 3DPC [107,108,109,110]. Oh and Choi [111] investigated the use of SAPs in 3DPC, focusing on the effects of the SAP dosage and particle size and the presence of SPs on the rheological behaviors of cement mortars. Their findings showed that although SAPs influenced the mortars’ properties similarly at low and high water–cement ratios when SPs were added, the rheological behavior varied over time and shear conditions. SAPs promoted structuring in mixtures without SPs, but in mixtures with SPs, the effect was reversed because of water migration dynamics associated with the SAPs. SAPs can serve as a promising rheology modifier for the development of 3D-printable cementitious materials [112].

In conclusion, rheological properties are influenced by several factors, including the type and dosage of SAPs, compatibility with other admixtures, and external conditions, such as temperature. SAPs have shown significant potential to modify and enhance concrete’s flow behavior, particularly in specialized applications, like 3D printing. However, the mechanisms governing the interaction between SAPs and the cement matrix and their impacts on key properties, such as the yield stress, plastic viscosity, and thixotropy, require further investigation. A deeper understanding of these interactions will enable the optimization of SAP formulations for various applications, ensuring enhanced performance and workability in both conventional and advanced cementitious systems.

## 4. Nanomaterials and Fibers

Nanoparticles have been increasingly incorporated into cement-based materials in recent years, aiming to enhance various properties, such as the hydration heat, workability, mechanical strength, water absorption, and durability. These nanomaterials also significantly alter the rheological properties of concrete, commonly resulting in reduced slump flow, increased viscosity, enhanced shear yield strength, and shortened setting times. The primary mechanism believed to drive these modifications is the increased surface area of the nanoparticles, which greatly impacts the behaviors of cementitious materials under various conditions. Furthermore, the use of fibers, such as natural fibers and synthetic fibers, in cement-based materials significantly influences their rheological properties, primarily by affecting the workability and flow behaviors. This chapter will further explore how nanoparticles and fibers influence the rheological properties of concrete.

### 4.1. Synthesized Nanomaterials

Synthesized nanomaterials refer to engineered materials, with nanometer-sized structures, that are specifically designed and produced through chemical or physical processes to achieve desirable properties. These materials include nano-silica (NS), nano-clays (NCs), carbon nanofibers (CNFs), and graphene oxide (GO), among others. Their distinct characteristics, such as large surface areas, reactivities, and abilities to interact with the cement matrix at a microscopic level, make them highly effective in modifying the rheological properties of cement-based materials. The incorporation of these synthesized nanomaterials significantly enhances key parameters, like the yield stress and thixotropy, which are crucial for improving the fresh and mechanical performance of concrete.

Lavergne et al. [113] demonstrated that NS particles could be effectively used as thickening agents in cement paste, although they also noted that the resultant slump reduction could affect the fluidity required for the concrete. Furthermore, Zhang et al. [114] studied the effects of colloidal NS with varying particle sizes on the flowability of cement pastes, revealing that the yield stress increased by 82.6%, 62.1%, and 38.2%, respectively, while plastic viscosity increased by 53.0%, 55.1%, and 57.3%. The observed increases in the yield stress were more pronounced than those in the plastic viscosity, attributed to the degree of colloidal NS flocculation, which restricts a part of the free water under ionic conditions, becoming more severe as the particle size decreases. Mohammed et al. [115] investigated the flow behaviors of cement pastes with NS under different temperature conditions, finding that the addition of 1% NS increased the shear stress limit from 15% to 52.7%. They determined that the percentage of the NS had the most significant impacts on the improvements of the initial shear stress, maximal shear stress, and plastic viscosity of the cement-based materials. Because of the impact of the NS on flocculation during the hydration of cement particles, it can increase the yield stress and plastic viscosity of the cement paste, significantly affecting its rheological properties. Therefore, it is necessary to adjust the percentage of the NS addition according to the rheological requirements of the paste to ensure that it meets the desired rheological performance.

Building on the understanding of NS’s significant impacts on rheological properties, it is pertinent to explore the effects of other nanomaterials, such as NCs, on cement-based materials. NCs have been widely incorporated into concrete because of their ability to significantly enhance the static yield stress over time. Their addition is especially valued for balancing the pumpability and buildability of concrete by increasing thixotropy, making them crucial for improving the overall handling and structural properties of materials [116]. A comparative analysis of the rheology and flowability of pastes blended with four types of NCs, including attapulgite, bentonite, and sepiolite (in both powder form and dispersed in water), showed that all the NCs reduced the flowability of the paste and modified its rheological properties. The addition of the sepiolite resulted in higher values of the yield stress and viscosity. The yield stresses of the pastes with the sepiolite and 0.35 w/b increased from 8.3 to 28.9 Pa, and the viscosities increased from 0.57 to 0.65 Pa·s. At 0.45 w/b, the yield stresses and viscosities of the pastes with the sepiolite increased by 134% and 267%, respectively [117]. Qian et al. [118] studied the compatibility between nano-clays and PCE in cement materials and their effects on rheology, finding that NCs increased the dynamic yield stresses of fresh cement pastes both with and without PCE addition while also enhancing thixotropy. The addition of NCs to cement pastes, particularly in high-PCE scenarios, significantly agglomerates the microstructure and elevates the thixotropic index.

The addition of NCs possesses enormous potential in the 3DPC field. Panda et al. [119] modified cement mortar with NCs for 3D printing to enhance buildability properties and reduce layer deformation. Their study indicated that NCs increase the static yield stresses of high-volume fly ash mortars, without significantly affecting the apparent viscosity, because of particle re-flocculation and enhanced thixotropy. The 0.5% NC mix had a higher static yield stress and almost the same apparent viscosity when compared to those of the control mix. The effect of the NCs on thixotropy was observed in terms of improvements in yield stress, without significantly affecting the viscosity. This behavior is highly advantageous in concrete-printing applications, where there is a demand for materials with high yield stress and low viscosity properties [120]. In addition, Zhang et al. [121] developed a novel 3DPC ink that exhibits good fluidity during movement and stable standing behavior in a static state by incorporating NCs and SF. The mix proportions featured a water-to-binder ratio of 0.35 by mass, with NCs serving as a partial cement substitute at a content of 2 wt.%. The rheological results of the fresh 3DPC indicated significant improvements in the yield stress and, particularly, thixotropy in mixes containing NCs. Liu et al. [122] explored the influences of ultrafine powders (NCs, nano-calcium carbonate (nano-CaCO_3_), and marble powder) on the properties of the lubrication layer in highly flowable concrete. Their findings indicated that a 1% replacement of the cement with NCs significantly increased the yield stress and plastic viscosity of the concrete. A similar replacement with nano-CaCO_3_ also increased these parameters, though to a lesser extent. Moreover, as the shearing time was extended, the yield stress and plastic viscosity of the unpumped concrete containing ultrafine powders increased. However, the viscosity constant of the lubrication layer and the pumping pressure for all the mixes, except those with 1% NCs, tended to decrease.

Carbon-based nanomaterials are increasingly incorporated into concrete to enhance its toughness and capacity for crack control. Studies have shown that the large specific surface areas of CNFs significantly impact the rheological properties of cement pastes [64]. The addition of CNFs leads to changes in the viscosity and yield stress of ultrahigh-performance concrete (UHPC) [123]. Wang et al. [124] investigated cement composites mixed with well-dispersed CNFs, examining the rheological properties of the cements with varying dosages of CNFs and water-to-cement (W/C) ratios. Their findings indicated that as the CNF dosage increased, the large amount of free water adsorbed on the CNFs’ surface made the cement pastes more viscous, thereby enhancing the flow resistance, yield stress, and plastic viscosity. Additionally, they noted that with an increasing W/C ratio, the particle concentration decreased and the particle spacing enlarged. This reduction in the zeta potential between the particles subsequently increased the liquidity of the cement composites. The impacts of CNFs on the rheological properties of 3D-printed cement-based materials have also been explored, with the results indicating that the rheological performances of mortar mixtures containing CNFs are improved. These improvements can be attributed to CNFs’ ability to form strong interfacial bonds with the cement matrix and act as nucleation sites for hydration reactions, thereby increasing the matrix’s yield stress [125]. The incorporation of natural fibers into cementitious composites has also been a topic of increasing research, as these fibers can significantly influence the rheological properties of the material. Studies on bio-based fibers, such as those derived from *Posidonia oceanica*, show that the addition of fibers to cement mixtures alters key rheological parameters, including the viscosity, yield stress, and thixotropy. Natural fibers can enhance the workability and reduce the segregation of cementitious mixtures, improving the overall stability of the mix [126].

The impact of GO on the rheological behavior of cement-based materials has been extensively documented. The use of GO significantly influences the flow behavior and flocculation processes within cement pastes [127,128,129,130]. GO can drastically increase the viscosities of cement-based materials [131]. Wang et al. [128] investigated the differences in the fluidity of cement pastes when mixed with GO alone or GO encapsulated in silica fume. They observed increases in both the yield stress and plastic viscosity with the addition of the GO. Further detailed investigations revealed that both the plastic viscosity and yield stress surged significantly as the GO concentration increased, particularly when it exceeded 0.01 wt.%. As the GO content increased, the cement pastes also exhibited enhanced thixotropic behavior, indicating that GO contributes to the formation of flocculated structures within the pastes. This highlights the influence of GO on the microstructural dynamics of the cement matrix. The distinct rheological properties modified by the appropriate dosage of GO can be harnessed for various construction applications. Its ability to enhance the viscosity and yield stress is essential for achieving the desired structural properties, making GO a valuable additive for developing advanced concrete formulations. This broad potential makes GO well suited for enhancing the performance of concrete.

In conclusion, the incorporation of synthesized nanomaterials, such as NS, NCs, CNFs, and GO, significantly modifies the rheological properties of cement-based materials, improving stability, workability, and performance. These nanomaterials offer considerable potential for enhancing concrete formulations, particularly in applications requiring precise control over rheology and improved durability.

### 4.2. Nanomaterials Produced from Industrial Byproducts

In addition to synthesized nanomaterials, several nanomaterials produced from industrial byproducts have also been explored for their potential to modify the rheological properties of cement-based materials. These nanomaterials not only improve the performance of concrete but also offer a sustainable approach for utilizing waste materials.

Variations in the particle shape, size, and size distribution; porosity; and chemical composition were all found to significantly influence the rheological properties of cement pastes, thereby impacting the performance of concrete. Gunnelius et al. [132] analyzed the effects of different types of functional fillers on the rheological properties of cement pastes and found that even the additions of low amounts of porous titania particles significantly increased the cohesive energy of the pastes. Furthermore, Li et al. [133] investigated the impacts of different sizes and crystal phases of nano-TiO_2_ (NT) on the rheological behaviors of cement pastes. The NTs significantly affected the rheological parameters of the cement pastes when the NT content exceeded 0.5 vol%. The yield stress and minimum viscosity of the cement pastes varied with the NT particle size by altering the total surface area of the system. Different crystal phases of NT also showed varied effects on the rheological parameters of the cement pastes, with increases in rheological parameters being more substantial for rutile NT than for anatase NT because of its higher average surface energy.

Senff et al. [134] conducted studies on the rheological properties of cement pastes and mortars by incorporating NS and NT. Their research demonstrated significant increases in the torque, yield stress, and plastic viscosity in mortars augmented with these nano-additives, effectively reducing the open testing time required in rheological assessments. Peng et al. [135] evaluated the rheological behaviors of cement–fly ash pastes that included nanomaterials, such as NS, nano-CaCO_3_, and nano-alumina (nano-Al_2_O_3_). Their findings indicated that both the type and concentration of the nanomaterials exerted substantial effects on the rheological properties of the mixtures. Specifically, the incorporation of NS and nano-Al_2_O_3_ led to increased yield stress and plastic viscosity while simultaneously decreasing the rheological index of the cement–fly ash pastes. Notably, when the contents of NS and nano-Al_2_O_3_ reached 2 wt.%, the rheological index dropped below 1, signifying a transition in the rheological behavior from shear thickening to shear thinning.

Expanding beyond conventional nanoparticles, Montes et al. [136] explored the effects of a novel nanoparticle, cellulose nanocrystals (CNCs), on the rheology of pastes. Their research revealed that higher dosages of CNCs, particularly as the water-to-cement ratio increased, resulted in the maximum reduction in the yield stress. Moreover, at lower dosages (less than 0.2%), the CNCs acted similarly to a water-reducing admixture by reducing the yield stress by up to 54%. In contrast, at higher dosages (greater than 0.5%), the CNCs increased the yield stress of the pastes, mirroring the behavior of certain VMAs. This variability in behavior underscores the potential of CNCs to be tailored for specific rheological adjustments in concrete applications, depending on the required properties of the pastes.

A summary of recent findings on the rheological modifications imparted by various nanomaterials is presented in Table 2, providing comparative insights into the specific effects of nanomaterials on key parameters, like yield stress, viscosity, and thixotropy, across different cementitious systems. This chapter thoroughly explored the significant impacts of nanomaterials, including, but not limited to, NS, NC, CNFs, and GO, on the rheological properties of cement-based materials. It detailed how these nanoparticles distinctly enhance properties such as viscosity, yield stress, and thixotropy. These improvements are crucial for advancing the performance, workability, and structural integrity of concrete, highlighting the transformative potential of nanotechnology in modern construction practices.

### 4.3. Fibers

The incorporation of fibers into cement-based materials significantly influences their rheological properties, primarily by affecting the workability and flow behavior. Studies [128,129] have shown that adding fibers often leads to a reduction in the workability because of the increased resistance to flow. For example, Huynh et al. [130] indicate that the inclusion of synthetic fibers in cement composites can decrease the workability of fresh concrete, necessitating adjustments in mix designs to achieve the desired performance. This reduction in the workability is attributed to the fibers’ tendency to impede the movement of cement particles, thereby increasing the mixture’s viscosity. Das et al. [131] reported that the use of a 2% concentration of hybrid fibers significantly lowered the workability of the fiber–concrete mixture compared to that of the control concrete mixture.

Moreover, the types and dosages of fibers play crucial roles in determining the extent of their impacts on rheological properties. High-performance fiber-reinforced cementitious composites, which utilize specific fiber types and proportions, have been developed to enhance ductility and tensile strength without severely compromising workability. For example, in one study, the rheology of the cement paste determined that the viscosity of the cement paste was related to the aspect ratio of the coconut fibers added, as well as to the percentage of the addition [132]. The yield stress and the apparent viscosity values were below the control sample’s corresponding values, providing better handling of the cement paste with the coconut fibers. The minimum yield stress was observed for AR = 7.143 and at a 2.5 wt.% addition [132]. However, achieving the optimal balance between improved mechanical properties and manageable rheological behavior requires the careful selection and proportioning of fibers. Excessive fiber content or inappropriate fiber types can lead to issues such as fiber clumping and non-uniform distribution, further deteriorating the workability of the cementitious mix.

## 5. Recommendations for Future Research

This review underscores the importance of optimizing the rheological properties of cement-based materials through the strategic integration of SCMs, chemical admixtures, nanomaterials, and internal curing agents. By systematically analyzing the effects of these materials on the properties of both fresh and hardened properties of concretes, this paper provides a comprehensive understanding of how these additives can work together to improve the performance, sustainability, and durability of concrete. However, future research should focus on the following aspects.

Although this review sheds light on the significant progress made in optimizing the rheological properties of materials, several key areas of future research warrant attention:

(1) The Synergistic Effects of Multiple Materials: Although much progress has been made in understanding the individual effects of SCMs, chemical admixtures, and nanomaterials, future research should focus on the synergistic interactions between these materials [133,134]. The combined effects of different additives on rheological and mechanical properties have not been sufficiently explored [135]. By understanding how these materials interact at both the macroscopic and microscopic levels, it will be possible to develop optimized formulations that maximize the benefits of each material while minimizing their limitations. A systematic approach for studying these synergies will allow for the design of concrete mixes with enhanced workability, stability, and durability, meeting the demands of advanced construction techniques;

(2) The Integration of Machine Learning and Artificial Intelligence: The application of machine learning (ML) and artificial intelligence (AI) presents a promising direction for future research in concrete formulation design [136,137]. By leveraging large datasets and advanced algorithms, ML models could predict the effects of various combinations of additives on concrete’s rheology and mechanical properties. For example, machine-learning algorithms, like XGBoost, or deep-learning models could be employed to optimize concrete mixtures in real time, providing insights into material interactions, performance, and sustainability [138]. Incorporating AI into the concrete mix design process can facilitate more efficient, data-driven decisions, leading to the development of smarter and more adaptive concrete formulations for a wide range of construction applications [139];

(3) Sustainability and Eco-friendly Additives: As sustainability continues to be a major focus in construction, the development of eco-friendly additives and materials for concrete is becoming increasingly important [140]. Future studies should explore alternative, waste-derived, or bio-based SCMs that not only reduce the carbon footprint of concrete production but also improve its overall performance. The exploration of recycled materials, such as waste glass, fly ash derived from alternative sources, and agricultural byproducts, as well as their roles in enhancing the rheological and mechanical properties of concrete, will be essential for achieving greener and more sustainable construction practices [141,142,143]. Research into sustainable additives and their long-term environmental impacts will be the key for reducing the construction industry’s environmental footprint;

(4) Advanced Characterization Techniques and Microstructural Analysis: A deeper understanding of the interactions between additives and the cement matrix requires the use of advanced characterization techniques. Techniques such as in situ Raman spectroscopy, in situ small-angle X-ray scattering, and nano computed tomography can provide detailed insights into how additives influence the microstructure of concrete, including hydration and the formation of hydration products. This research will help to elucidate the mechanisms by which different materials affect the rheological behavior and long-term durability. Understanding these microstructural changes will be essential for developing tailored, high-performance concrete formulations that meet the specific demands of construction and environmental conditions;

(5) Customization for Emerging Construction Technologies: With the advent of new construction technologies, such as 3DPC, UHPC, and self-healing concrete, the rheological properties of concrete need to be optimized to meet specific requirements. For instance, 3D printing requires concrete to be highly stable while maintaining excellent flow properties for smooth deposition [144]. Similarly, UHPC demands specific rheological and mechanical properties for use in extreme environments [145]. Future research should focus on optimizing concrete for these emerging applications, ensuring that it can meet the specialized needs of modern construction methods while maintaining high performance and durability.

## 6. Conclusions

This review highlights key strategies for optimizing the rheological properties of cement-based materials through the integration of SCMs, chemical admixtures, nanomaterials, and internal curing agents. These additives play critical roles in modulating essential rheological parameters, such as the yield stress, plastic viscosity, and thixotropy, which directly impact the workability, stability, and overall performance of concrete in both fresh and hardened states. One of the key contributions of this review is its systematic approach for categorizing these materials based on their properties and their effects on the rheology of cement-based materials. This comprehensive understanding is crucial for advancing concrete formulations and tailoring mixes for specific construction needs while reducing the environmental impacts of cement production. The following conclusions are presented:

1. SCMs (e.g., FA, GGBFS, and SF) reduce clinker dependency while enhancing rheological performance through distinct physical–chemical mechanisms. The spherical morphology of FA improves flowability, whereas GGBFS and SF optimize particle packing and hydration kinetics. Emerging SCMs, like metakaolin, further demonstrate the potential to decouple sustainability goals from performance tradeoffs;

2. Chemical admixtures enable precise rheological control by targeting specific challenges: SPs mitigate particle agglomeration through electrostatic/steric effects, while VMAs counteract segregation via controlled viscosity modulation. The compatibility between SPs and VMAs underscores the importance of molecular-level design in achieving concurrent flowability and stability;

3. SAPs exemplify the dual functionality of internal curing agents, where their water retention capacity directly modifies the hydration dynamics and rheological evolution. The interaction between SAPs and admixtures highlights the need for the temporal optimization of rheological behaviors in complex curing environments;

4. Nanomaterials (NS, NCs, CNFs, and GO) bridge the gap between macro-rheology and microstructural engineering. NS and NCs enhance the thixotropy and yield stress through nucleation and physical network formation, whereas CNFs and GO reinforce interfacial transition zones. These effects position nanomaterials as pivotal components in advanced applications, like 3D-printed concrete.

## Figures and Tables

**Table 2:** Impacts of various nanomaterials on the rheological properties of cement-based materials.

Type	Dosage	Rheological Impact(s)	Key Finding(s)	Study
NS	1.5%, 3%, and 5% by the weight of the binder	Acts as a thickening agent, reducing the slump and potentially affecting the fluidity.	It is an effective thickening agent but reduces the fluidity, emphasizing the need for the proper dosage.	[113]
	A mass fraction of 5% of the cement	The yield stress increases by 82.6%, 62.1%, and 38.2%, while the plastic viscosity rises by 53.0%, 55.1%, and 57.3%.	Nanoparticles increase the yield stress significantly because of flocculation, affecting free water’s mobility.	[114]
	1% NS	The shear stress limit increased from 15% to 52.7%.	The percentage of the NS strongly impacts the shear stress and plastic viscosity.	[115]
NCs	0, 0.1, 0.3, and 0.5% by the mass of the binder	With a 0.5% NC addition, the static yield stress increased from 19 Pa to 58.2 Pa.	The cohesion and static yield stress increased with increasing NC addition.	[116]
	2% by the cement weight for the four NCs (attapulgite, bentonite, and sepiolite)	The yield stress and viscosity increased, particularly with the sepiolite addition.	They enhanced the static yield stress and thixotropy and were ideal for pumpability and buildability.	[117]
	0.5% NCs	Increased the static yield stress without affecting the apparent viscosity.	Effective in 3DPC, providing high yield stress and low viscosity for improving the layer stability.	[119]
Nano-CaCO_3_	1% replacement of the cement	Increased the yield stress, plastic viscosity, and thixotropic behavior	Improved flow characteristics while maintaining stability	[122]
	Replaced 0%, 2%, and 4% by mass of the cement	Using nano-CaCO_3_ increased the shape retention factor from 0.87 to 0.97.	The addition of nano-CaCO_3_ improved the rheology.	[137]
NT	0, 0.1%, 0.3%, 0.5%, 0.7%, and 1.4 vol% of the cement	Increased the cohesive energy and rheological parameters (yield stress and viscosity) depending on the particle size and crystal phase (rutile > anatase)	Rutile NT increased the rheological parameters because of its higher surface energy, highlighting the impacts of the particle size and structure.	[133]
	0–12 wt.%	The addition of the NT significantly increased the values of the torque and yield stress, corresponding to a decrease in the open testing time.	Using SP at varying levels, depending on the concentration of the NT, resulted in additional performance improvements and distinct variations in hydration kinetics.	[134]
CNCs	Addition rates from 0.023 vol% to 3.311 vol% relative to the dry cement	Low dosages reduced the yield stress by 54%; high dosages increased the yield stress by over 10 times the original value.	Versatile behavior, with water-reducing properties at low dosages, making CNCs adaptable to varied rheological requirements.	[136]
CNFs	Variable CNF dosages (0, 0.05%, 0.1%, 0.2%, 0.3%, 0.4%, and 0.5%)	Higher viscosity, yield stress, and flow resistance with increased CNF content; the increased rate of the yield stress ranged from 167% to 11,867%.	Strong interfacial bonds and nucleation sites from CNFs increased the yield stress, which is ideal for high-performance applications.	[124]
GO	0.01, 0.03, and 0.05 wt.% of the cement	The effects of the GO increased the yield stress and plastic viscosity and enhanced thixotropy and flocculation.	GO promotes microstructural flocculation, with significant effect on fresh and long-term performance	[128]

## Data Availability

No new data were created or analyzed in this study.
